# Overexpression of microRNA-21 strengthens stem cell-like characteristics in a hepatocellular carcinoma cell line

**DOI:** 10.1186/s12957-016-1028-9

**Published:** 2016-10-28

**Authors:** Jinghang Jiang, Peipei Yang, Zhe Guo, Rirong Yang, Haojie Yang, Fuquan Yang, Lequn Li, Bangde Xiang

**Affiliations:** 1 Department of Hepatobiliary Surgery, Tumor Hospital of Guangxi Medical University, Nanning, 530021 Guangxi Zhuang Autonomous Region China; 2 Department of General Surgery, The Second People’s Hospital of Jing Men, Jingmen, 448000 Hubei Province China; 3 Department of Thyroid and Breast Surgery, The Central Hospital of Wuhan, Wuhan, 430041 Hubei Province China; 4 Department of Immunology, School of Preclinical Medicine, Biological Targeting Diagnosis and Therapy Research Center, Guangxi Medical University, Nanning, 530021 Guangxi Zhuang Autonomous Region China; 5 Department of General Surgery, The First People’s Hospital of Changde, Changde, 415000 Hunan Province China

**Keywords:** Hepatocellular carcinoma, Liver cancer stem cells, microRNA-21, Cancer stem cell, Retroviral vector

## Abstract

**Background:**

Liver cancer stem cells (LCSCs) have been shown to express higher levels of microRNA-21 (miR-21). Here, we examine the possible contributions of miR-21 to the phenotype of LCSCs in culture and in xenograft tumors in nude mice.

**Methods:**

The hepatocellular carcinoma cell line MHCC-97H was stably transformed with a retroviral vector to establish cells overexpressing miR-21, while a cell line transformed with empty vector served as a negative control. RT-PCR and Western blotting were used to evaluate the effects of miR-21 overexpression on the expression of various LCSC markers, a Transwell assay was used to assess the effects on cell migration and invasion, and a spheroid formation assay was used to examine the effects on clonogenesis. The effects of miR-21 overexpression were also examined in tumors in nude mice.

**Results:**

An MHCC-97H cell line was constructed that stably overexpresses miR-21 at 7.78 ± 1.51-fold higher levels than the negative control cell line. Expression of the LCSC markers CD13, Ep-CAM, CD90, and OCT4 was significantly higher in the miR-21-overexpressing cell line than in the negative control at both mRNA and protein levels. The overexpressing cell line formed larger, tighter, and more numerous spheroids. Overexpression of miR-21 was associated with greater cell migration and invasion. Tumors of overexpressing cells in nude mice had a significantly larger mean volume after 34 days of growth (773.62 ± 163.46 mm^3^) than tumors of negative control cells (502.79 ± 33.94 mm^3^, *p* = 0.048), as well as greater mean weight (0.422 ± 0.019 vs. 0.346 ± 0.006 g, *p* = 0.003).

**Conclusions:**

Overexpression of miR-21 strengthens the phenotype of LCSCs, facilitating invasion, migration, and tumorigenesis in hepatocellular carcinoma.

## Background

Hepatocellular carcinoma (HCC) is one of the most common malignant tumors and a leading cause of cancer-related deaths worldwide [1], making it a major health burden [2]. Challenges in early HCC diagnosis mean that most cases are detected too late to be curable. Surgical resection remains the preferred treatment [3, 4], but postoperative recurrence is high [5]. Therefore, understanding how HCC recurs may allow the design of treatments or interventions to reduce it.

Cancer stem cells (CSCs), also referred to as “side populations” because they are low-abundance subpopulations in tumors, can initiate and sustain cell migration, invasion, and treatment resistance. CSCs are responsible for poor clinical outcome in tumor recurrence and metastasis [6, 7]. The malignancy of a tumor correlates directly with the proportion of CSCs that it contains [8]. CSCs are not sensitive to radio- or chemotherapy [9, 10], so they can evade treatment and survive to cause recurrence. Liver cancer stem cells (LCSCs), like other CSCs, are sub-populations of tumor cells capable of unlimited proliferation, self-renewal, and differentiation [11, 12]. According to a hypothesis advocated by many investigators [13], the chief culprit behind HCC recurrence is LCSCs [6, 7, 13]. Inhibiting LCSC proliferation and differentiation or even eradicating LCSCs entirely may prove critical for reducing HCC recurrence after hepatectomy [14, 15].

To move closer to this objective, researchers have developed methods to isolate LCSCs from HCC tumor tissue and HCC cell lines such as MHCC-97H. Researchers have screened for microRNAs (miRNAs) differentially expressed between LCSCs and HCC cell lines. In many cancers including HCC, miRNAs function as tumor suppressor genes and as oncogenes to modulate tumor cell proliferation, apoptosis, differentiation, and cell cycling [16]. In addition, miRNAs regulate self-renewal and multi-differentiation to determine stem cell fates [17–19]. In LCSCs, down- and upregulation of certain miRNAs influence LCSC characteristics such as clonogenicity and cell proliferation [19].

Recent studies have shown that microRNA-21 (miR-21) is expressed at higher levels in various CSCs than in the corresponding bulk tumor cells and that it influences CSC behavior [20]. Inhibiting miR-21 expression increases sensitivity to chemotherapy and inhibits colony-forming ability. LCSCs, like other CSCs, have been shown to express higher levels of miR-21 than bulk HCC cells [21], and studies in which LCSCs were transfected with an miR-21 mimic or inhibitor suggest that miR-21 drives HCC recurrence and metastasis [22]. These studies were performed on isolated LCSCs, leaving open the question of whether overexpression of miR-21 strengthens stem cell-like characteristics in a hepatocellular carcinoma cell line, which may provide an excellent in vitro system for studying processes that occur in postoperative HCC patients.

Therefore, in the present study, we generated an MHCC-97H cell line stably transfected with a retroviral vector encoding miR-21. We then analyzed in vitro the effects of constitutive miR-21 overexpression on the expression of various LCSC markers (CD13, Ep-CAM, CD90, OCT4) as well as on several LCSC functional characteristics (spheroid formation, invasion, migration). The cell surface markers CD13, Ep-CAM, and CD90 are often used to enrich for LCSCs [23–26], while OCT4 is a transcription factor essential for pluripotency and self-renewal of embryonic stem cells [27–29]. To complement these in vitro studies, we examined the effects of miRNA-21 overexpression on tumorigenicity in nude mice.

## Methods

### Cell culture

MHCC-97H and HEK 293 T cells were obtained from the Liver Cancer Institute of Zhongshan Hospital, Fudan University (Shanghai, China) and cultured in Dulbecco’s modified Eagle’s medium (DMEM; Gibco, USA) supplemented with 10 % fetal bovine serum (FBS; Gibco, USA), 100 units/ml penicillin, and 100 mg/ml streptomycin. Cultures were incubated at 37 °C in a humidified atmosphere containing 5 % CO_2_.

### Plasmid constructs

We cloned the miR-21 gene from 7702 liver cell genomic DNA (kindly provided by the Department of Immunology, Guangxi Medical University) using the following primers, which we designed using the Primer 5.0 software (Premier Biosoft International, USA): BamH1 forward primer, 5′-CGCGGATCCTTCTTGCCGTTCTGTAAGTGTT-3′; and SalI reverse primer, 5′-AGACGTCGAC TTCAAAACCCACAATGCAGCTTAG-3′. The primers were used in a polymerase chain reaction (PCR) to amplify a 589-bp product, which was cleaved with BamH1 and Sal1 and ligated into the appropriate cloning sites of the pBABE-puro retroviral vector (Cell Biolabs, USA). The resulting construct was named pBABE-puro-pre-miR-21.

### Retroviral particle production

HEK 293 T cells were plated in six-well plates and maintained for 24 h in DMEM with 10 % FBS. When cells were 60–70 % confluent, they were transfected with 1 μg of packaging plasmid PIK [30, 31] (kindly provided by the Department of Immunology, Guangxi Medical University), empty pBABE-puro vector, or pBABE-puro-pre-miR-21 using Lipofectamine 2000 (Invitrogen, USA) in OPTI-MEM I medium (Invitrogen) according to the manufacturer’s instructions. After the 5-h incubation, the medium was replaced with DMEM containing 10 % FBS without antibiotics. Cells were incubated for another 48 h, and the medium containing retroviral particles was harvested, filtered through a 0.22-μm filter (Corning, USA) into a Falcon tube, and stored at −80 °C.

### Stable cell line construction

MHCC-97H cells were seeded into six-well plates and allowed to grow to 70–80 % confluence. Then, the cells were infected with retroviral particles lacking miR-21 (negative control) or containing miR-21 in the presence of polybrene (8 μg/ml, Sigma-Aldrich, USA) and incubated for 24 h at 37 °C. The culture medium enriched in retroviral particles was harvested and used to infect fresh plates of 70–80 % confluent MHCC-97H cells, which were incubated for another 24 h. The medium was replaced with DMEM containing 10 % FBS and 1 μg/ml puromycin (Sigma) without other antibiotics, and cultures were incubated for 2 weeks. Medium was replaced every 3 days. Finally, the medium was replaced with DMEM containing 10 % FBS, 100 units/ml penicillin, 100 mg/ml streptomycin, and 500 ng/ml puromycin, and cultures were incubated for 2 months. The medium was replaced every 3 days.

### Quantitative real-time reverse transcription-PCR (RT-PCR) to measure expression of miR-21 and CSC marker genes

Total RNA was extracted from cells using Trizol (Invitrogen) according to the manufacturer’s protocol. Primers for reverse transcription were the following: miR-21, 5′-CTCAACTGGTGTCGTGGAGTCGGCAATTCAGTTGAGTCAACAT-3′; and U6, 5′-CTCGCTTCGGCAGCACA-3′. The reverse transcription reaction was carried out at 4 °C for 20 min, 16 °C for 30 min, 42 °C for 30 min, and 85 °C for 5 s, followed by a hold at 4 °C.

Reverse transcription of CSC marker genes was carried out using the PrimeScript® RT Kit (Takara Biotechnology, Dalian, China), followed by quantitative PCR in a 7300 Real-Time PCR System (Applied Biosystems, USA) using SYBR® Premix Ex Taq™ II (Takara Biotechnology). The primers used in these reactions are listed in Table 1. Levels of miR-21 were normalized to levels of U6, while levels of CSC marker mRNAs were normalized to levels of glyceraldehyde-3-phosphate dehydrogenase (GAPDH) mRNA. All results were calculated using the 2[−ΔΔC(T)] method. All experiments were performed in triplicates.

**Table 1:** Primers used for reverse transcription and amplification of genes related to liver cancer stem cells

Gene	Primer sequence(5′→3′)
miR-21	F: ACTCAGCTGG TAGCTTATCAGACTGATG
	R: TGGTGTCGTGGAGTCG
U6	F: CTCGCTTCGGCAGCACA
	R: AACGCTTCACGAATTTGCGT
CD13	F: TTCAACATCACGCTTATCCACC
	R: AGTCGAACTCACTGACAATGAAG
Ep-CAM	F: ATAACCTGCTCTGAGCGAGTG
	R: TGCAGTCCGCAAACTTTTACTA
CD90	F:ATGAAGGTCCTCTACTTATCCGC
	R: GCACTGTGACGTTCTGGGA
OCT4	F: GTGTTCAGCCAAAAGACCATCT
	R: GGCCTGCATGAGGGTTTCT
GAPDH	F: CTGGGCTACACTGAGCACC
	R: AAGTGGTCGTTGAGGGCAATG

*F* forward, *R* reverse

### Western blot analysis

Total cell lysates were prepared from the negative control cell line and cell line stably transfected with miR-21, fractionated on SDS-PAGE and transferred to PVDF membranes (Millipore). After blocking the membranes in phosphate-buffered saline (PBS) containing 0.1 % Tween 20 (PBST) and 5 % non-fat dry milk for 2.5 h at room temperature, the PVDF membranes were probed at 4 °C overnight with rabbit antibodies (Abcam, UK) against CD13 (1:2,000), Ep-CAM (1:2,000), CD90 (1:1,000), and OCT4 (1:2,000). Then, membranes were washed with PBST three times and incubated for 2 h at room temperature with horseradish peroxidase (HRP)-conjugated secondary antibody (1:2,000; Abcam). After washing the membranes three times with PBST, the proteins were detected using enhanced chemiluminescence (Thermo, USA) and quantified using ImageLab 5.0 (Bio-Rad, USA). Protein levels were normalized to the amount of GAPDH detected on the same blot. Representative data from three independent experiments are shown.

### Spheroid formation assay

To measure spheroid formation by stable cell lines, negative control cells and cells stably transfected with miR-21 were mixed with serum-free DMEM/F12 medium containing B27 supplement (1:50; Invitrogen), 20 ng/mL epidermal growth factor (Invitrogen), and 20 ng/mL basic fibroblast growth factor (Invitrogen) and plated in six-well dishes (2000 cells per well). Cells were incubated for 14 days, and the medium was replaced every 3 days. Spheroids were counted and photographed under a microscope (Nikon, Tokyo, Japan). All experiments were performed in triplicates.

### Migration and invasion assays

Cell migration and invasion were analyzed using Transwell cell culture chambers with 8-μm pores (Corning, USA). The upper chamber was coated with Matrigel (Corning) for invasion assays, but not for migration assays. Cells (10^5^) were added to the upper chamber without FBS, while lower chambers were immersed in medium containing 10 % FBS as a chemoattractant; the culture chambers were then incubated for 24 h at 37 °C. Cells were removed from the upper chamber using cotton swabs, and the filters were stained with methanol for 5 min at 37 °C, then fixed with Giemsa’s solution (Solarbio, China) for 10 min at 37 °C. Numbers of migrating and invasive cells were counted in five randomly selected fields on each filter. Representative data from three independent experiments are shown.

### Tumorigenicity in vivo

Animal studies were performed according to the Guidelines of Intramural Animal Use and were approved by the Animal Ethics Committee of Guangxi Medical University. Negative control cells or cells stably transfected with miR-21 (1 × 10^7^) were suspended in a 1:1 (*v*/*v*) mixture of 100 μl PBS and Matrigel (BD, USA) and injected subcutaneously into both sides of five male BALB/C nude mice aged 5–6 weeks (Laboratory Animal Center, Guangxi Medical University). Tumor length and width were examined every 3–5 days in a specific pathogen-free laboratory using a vernier caliper. At 34 days after injection, nude mice were given an intraperitoneal injection of chloral hydrate, and then killed by cervical dislocation. Tumors were weighed, and tumor volumes were calculated using the equation: volume (mm^3^) = (length × width^2^)/2.

### Statistical analysis

Statistical analysis was performed using SPSS 19.0 (IBM, USA). Data were presented as mean ± standard deviation, and inter-group differences were assessed for significance using Student’s *t* test. All statistics should be two-tailed, and the threshold of significance was defined to be *p* < 0.05.

## Results

### An HCC cell line stably overexpressing miR-21

**Fig. 1:**
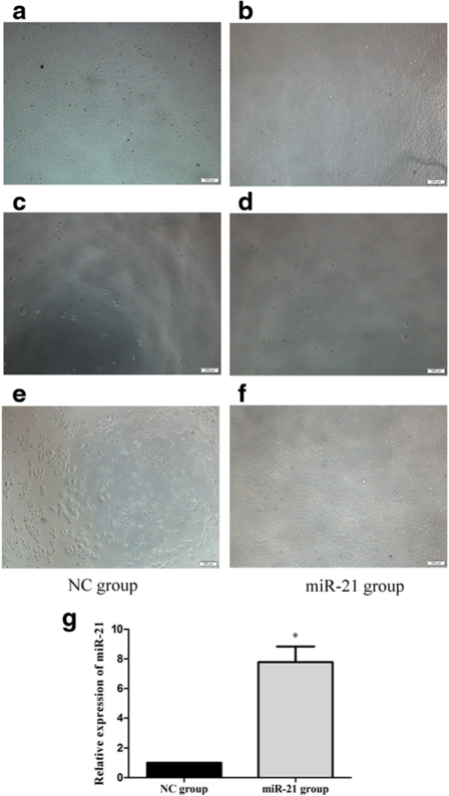
An MHCC-97H cell line stably overexpressing miR-21 was selected and cultured in the presence of puromycin (magnification, ×200). **a**, **b** Negative control (NC) cultures and cultures stably transfected with miR-21 were mock-transfected in the absence of retroviral particles. **c**, **d** Transfected cells were grown for 2 weeks in DMEM containing 1 μg/ml puromycin without other antibiotics. **e**, **f** The two cell lines were cultured for 2 months in DMEM containing 500 ng/ml puromycin without other antibiotics. **g** Relative expression of miR-21 was determined by RT-PCR. *p < 0.05, compared with NC

The analysis of PCR reactions on 1.0 % agarose showed the desired 589-bp miR-21 amplicon, which was digested with BamHI and SalI for insertion into the retroviral vector. The expression construct pBABE-puro-pre-miR-21 was transformed into *E. coli* DH5. Plasmid DNA was isolated from a panel of transformants and digested with BamHI/SalI to screen for the 589-bp insert. The resulting pBABE-puro-pre-miR-21 was used to create an MHCC-97H cell line stably overexpressing miR-21 (Fig. 1). RT-PCR analysis showed that miR-21 expression was 7.78 ± 1.51-fold higher in the miR-21-transfected cultures than in the negative control cultures (*p* < 0.05).

### miR-21 increased the expression of LCSC markers

**Fig. 2:**
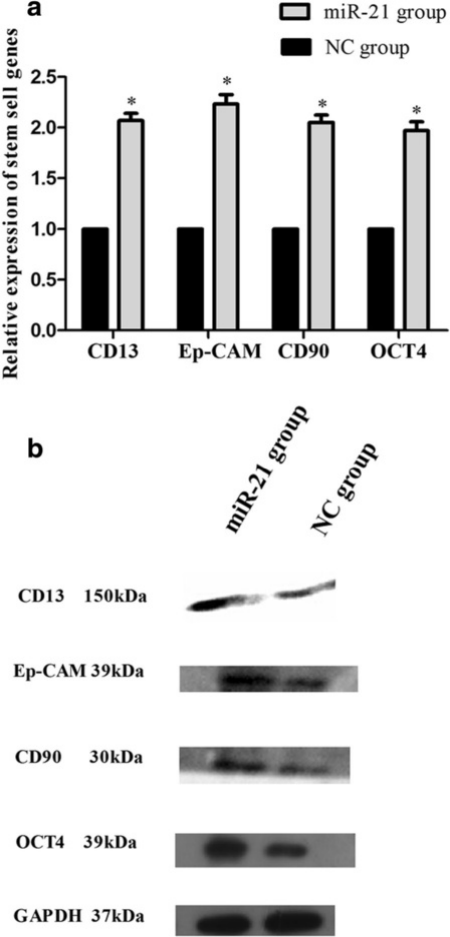
Expression of LCSC markers in an MHCC-97H cell line stably overexpressing miR-21 cells. **a** The levels of the corresponding mRNAs were determined using RT-PCR. **b** The levels of CD13, Ep-CAM, CD90, and OCT4 protein in the miR-21-overexpressing cell line and corresponding negative control (NC) line were measured using Western blotting. *p < 0.05, compared with NC

Cells stably overexpressing miR-21 contained approximately twofold more mRNA encoding four LCSC markers than in the negative control cells: for CD13, the difference was 2.07 ± 0.10-fold (*p* = 0.004); Ep-CAM, 2.23 ± 0.13 (*p* = 0.006); CD90, 2.01 ± 0.10 (*p* = 0.005); and OCT4, 1.97 ± 0.12 (*p* = 0.008; Fig. 2a). Expression of the four corresponding proteins was also significantly higher in miR-21-overexpressing cells (*p* < 0.05; Fig. 2b). These results indicate that miR-21 overexpression strengthened the LCSC phenotype.

### miR-21 increased clonogenicity

**Fig. 3:**
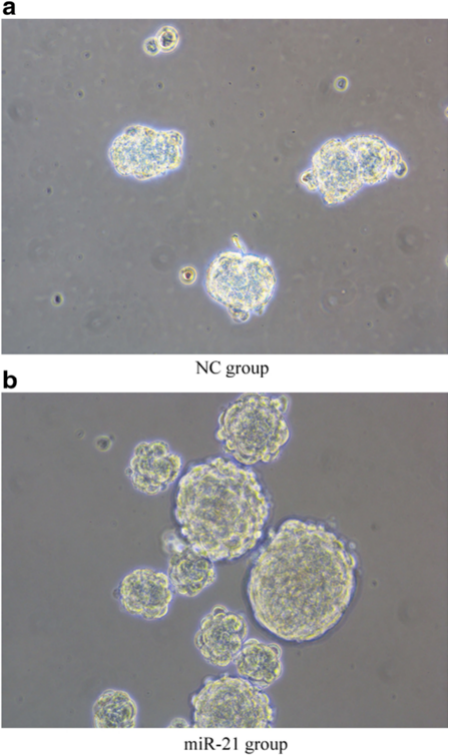
Overexpression of miR-21 promotes spheroid formation. **a**, **b** MHCC-97H cell line stably overexpressing miR-21 and the corresponding negative control (NC) cell line were induced to form spheroids for 14 days. The miR-21-overexpressing cell line formed larger, tighter, and more numerous spheroids

Negative control cells and cells stably overexpressing miR-21 formed loose aggregates that eventually coalesced into compact, tight, rounded spheres that grew over the 14-day incubation. Sphere morphology was analyzed in detail using light microscopy, which showed that miR-21-overexpressing cells formed tighter, larger, more numerous, and more confluent spheres (Fig. 3). These results indicated that higher expression of LCSC surface markers correlated with greater sphere-forming ability.

### miR-21 promoted migration and invasion

**Fig. 4:**
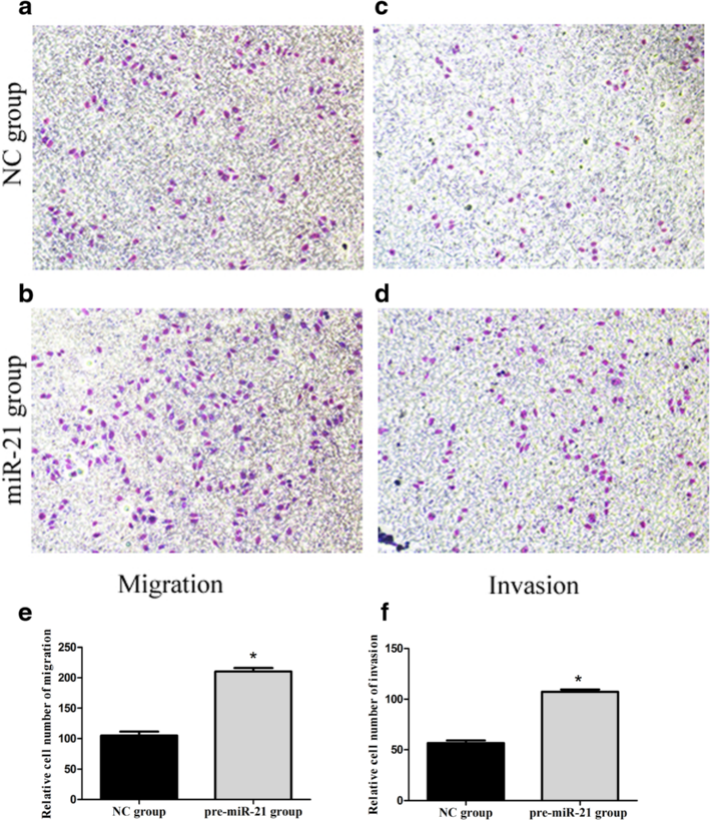
Overexpression of miR-21 promotes cell migration and invasion. **a**-**d** Transwell migration and Matrigel invasion assays were carried out using MHCC-97H cells stably overexpressing miR-21 or the corresponding negative control (NC) cells. **e**, **f** Numbers of migrating and invading cells counted in five fields randomly selected on the Transwell culture insert. *p < 0.001, compared with NC

In Transwell assays, miR-21-overexpressing cells showed greater migration and invasion ability than the negative control cells. The number of migrating cells measured in randomly selected fields of the Transwell culture insert was significantly higher with the miR-21-overexpressing cultures than the negative control cultures (210.3 ± 5.8 vs. 104.8 ± 6.5, *p* < 0.001), and the relative number of cells that invaded through the extracellular matrix coating was significantly higher for the miR-21-overexpressing cultures (107.3 ± 2.3) than for the negative control cultures (56.7 ± 2.5, *p* < 0.001; Fig. 4).

### miR-21 accelerated tumor growth in BALB/C nude mice

**Fig. 5:**
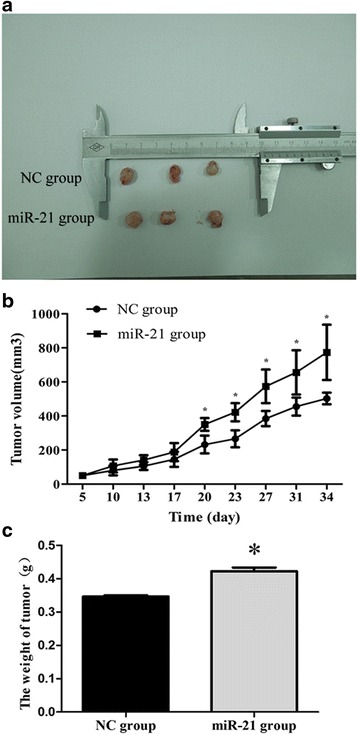
Overexpression of miR-21 promotes the growth of subcutaneous xenograft tumors in nude mice. **a** Representative xenograft tumors grown for 34 days using MHCC-97H cells stably overexpressing miR-21 or the corresponding negative control (NC) cells. **b** The tumor volume and **c** tumor weight were compared between the two stable cell lines. *p < 0.05, compared with NC

BALB/C nude mice were inoculated with negative control cells or cells stably transfected with miR-21, and tumors were allowed to grow. Tumors were visible after 5 days in both groups of mice, but tumors from cells overexpressing miR-21 grew significantly faster (Fig. 5b). On day 28 after inoculation, two mice died, and postmortem dissection showed them to have pulmonary metastases. Mean tumor volume at the end of the 34-day observation period was 773.62 ± 163.46 mm^3^ in the miR-21 group and 502.79 ± 33.94 mm^3^ in the negative control group (*p* = 0.048), while the corresponding mean weights were 0.422 ± 0.019 g and 0.346 ± 0.006 g (*p* = 0.003, Table 2 and Fig. 5c), respectively. Tumor pathology was verified by histopathology.

**Table 2:** Subcutaneous xenograft tumor weight at 34 days after tumor cell injection

Cell line	Weight, g (mean ± SD)	*P*
Negative control	0.346 ± 0.006	0.003
miR-21	0.422 ± 0.019	

## Discussion

LCSCs are thought to arise from hepatocyte de-differentiation or transformation from hepatic progenitor cells, and many investigators believe that LCSCs generate and maintain HCC and drive postoperative recurrence [15, 32, 33]. This implies that inhibiting and eliminating LCSCs may be key to reducing recurrence [34, 35]. Here, we confirm and extend previous work in isolated LCSCs by showing that overexpression of miR-21 in the HCC cell line MHCC-97H directly affects numerous functional characteristics of LCSCs, providing mechanistic insight into the events that may drive HCC recurrence and suggesting a potential therapeutic target.

Ep-CAM and CD90 are widely regarded as LCSC markers, and higher expression of these markers has been linked to greater risk of recurrence and shorter survival time in HCC [23, 24]. We found that miR-21 overexpression was associated with increases in expression of these markers and of the LCSC markers CD13 and OCT4. Previous work showed that miR-21 can promote tumor spread by upregulating the expression of phosphatase and tensin homolog (PTEN), programmed cell death 4 (PDCD4) protein, and reversion-inducing cysteine-rich protein with Kazal motifs (RECK) [22]. Future studies should aim to determine the full range of target genes affected by miR-21, since this may help clarify the complex literature on risk factors of HCC recurrence.

CD13, which helps pause LCSCs in G1/G0 phase of the cell cycle [25], has already proven a useful drug target: the combination of a CD13 inhibitor with the genotoxin fluorouracil (5-FU) led to significantly smaller tumor size than either drug on its own in a mouse xenograft tumor model [26]. CD13 is special among LCSC markers because its level changes during differentiation of LCSCs in culture [36]: upregulation of CD13 is associated with the epithelial-mesenchymal transition and with a reduction in apoptosis. It is also associated with the resistance of LCSCs to chemotherapy and more robust survival [37]. OCT4 plays a pivotal role in maintaining the multidirectional differentiation and self-renewal properties of embryonic stem cells [27–29]. OCT4 is highly expressed by cells highly enriched for CD90- and CD133-positive LCSCs, and its expression is tightly associated with chemotherapy resistance [38]. Coexpression of OCT4 and another “stem-ness” marker, Nanog, by HCC cells is associated with aggressive tumor behavior and worse clinical outcome [28]. OCT4 can promote CSC-like traits as well as the epithelial-mesenchymal transition in HCC cells; it exerts these effects by regulating the Stat3/Snail pathway [39]. Future work should examine whether OCT4 may be a reasonable therapeutic target for preventing HCC recurrence.

How miR-21 may promote the behavior of LCSCs, or of CSCs in general, remains unclear. Some mechanisms have been proposed [40]. One is that miR-21 acts within the tumor progenitor cells to modulate their self-renewal. Another is that miR-21 in non-progenitor tissue triggers tumor cell differentiation, leading to CSC production. A third is that miR-21 in non-progenitor cells produces growth factors that benefit CSCs.

Our observation that miR-21 overexpression increases the ability of MHCC-97H cells to invade and metastasize echoes a report that transfecting SMMC-7721 cells with an miR-21 analog markedly enhanced their invasion and metastasis abilities. Indeed, inhibiting miR-21 in MHCC-97H cells reduced these abilities [41]. Similarly, silencing trans-expressed miR-21 in isolated LCSCs reduced their migration and invasion [22].

Our observation that miR-21 overexpression leads to faster tumor growth in a nude mouse xenograft model is consistent with a report that inhibiting miR-21 in pancreatic CSCs substantially decreased the growth of pancreatic tumor xenografts [42]. In another study, colorectal cancer cells were stably transfected with miR-21 plasmid and subcutaneously injected into female SCID mice [43]. At 6 weeks after injection, tumors were larger in mice injected with cells stably transfected with miR-21 than in mice injected with the corresponding empty vector. That work further showed that miR-21 overexpression can induce the appearance of colorectal CSCs.

## Conclusions

The results of the present study suggest that miR-21 overexpression can upregulate CD13, Ep-CAM, CD90, and OCT4 expression. In addition, miR-21 strengthens LCSC characteristics, including spheroid formation, invasion, and migration. CSC marker expression may regulate the expression and activity of miR-21, thereby reinforcing the stem cell phenotype.

## Abbreviations

CSCs: Cancer stem cells
HCC: Hepatocellular carcinoma
LCSCs: Liver cancer stem cells
miR: microRNA
PDCD4: Programmed cell death 4
PTEN: Phosphatase and tensin homolog
RECK: Reversion-inducing cysteine-rich protein with Kazal motifs
RT-PCR: Quantitative real-time reverse transcription-PCR
